# Relationship between admission blood urea nitrogen levels and postoperative length of stay in patients with hip fracture: A retrospective study

**DOI:** 10.1097/MD.0000000000038918

**Published:** 2024-07-12

**Authors:** Teng-di Fan, Di-kai Bei, Qi Wang

**Affiliations:** a Department of Orthopedics, Ningbo Medical Center Lihuili Hospital, Ningbo, Zhejiang, China; b Department of Medical Record Office, Ningbo Medical Center Lihuili Hospital, Ningbo, Zhejiang, China.

**Keywords:** biomarker, blood urea nitrogen level, hip fracture, length of stay

## Abstract

To investigate the relationship between admission blood urea nitrogen (BUN) levels and postoperative length of stay (LOS) in hip fracture (HF) patients. This retrospective study retrieved related data from the MIMIC-IV database, of which the laboratory variables were taken preoperatively. The patients were divided into 4 groups according to the BUN quartile levels. After exploring the nonlinear relationship between BUN and LOS by generalized additive model, their connection was further analyzed using the generalized linear models, quantile regression models, and interaction analysis. Receiver operating characteristic curve analysis and decision curve analysis were performed to evaluate its value in predicting first intensive care unit admission and in-hospital mortality. Totally 1274 patients with HF were enrolled in the study. There was a nonlinear relationship between BUN and LOS (*P* < .05). Besides, BUN was an independent predictor for LOS after adjusting different covariates in 3 models (*P* < .05). Age served as a significant interactor in this relationship (*P* < .05). Moreover, receiver operating characteristic curve and decision curve analysis revealed the predictive value of BUN for intensive care unit admission and in-hospital mortality in HF. Admission BUN level as a cost-effective and easy-to-collect biomarker is significantly related to LOS in patients with HF. It helps clinicians to identify potential high-risk populations and take effective preventions before surgery to reduce postoperative LOS.

## 1. Introduction

Hip fracture (HF) is an essential medical condition related to adverse clinical outcomes such as hospitalization, disability, and mortality.^[1]^ The incidence of HF varies by 10-fold with the highest rate in Northern Europe, the moderate in the United States, and the lowest rate in Latin America and Africa.^[2]^ The aging populations worldwide may lead to an increased incidence of HF. The number of HF patients was 1.6 million in 2000, which is expected to be up to 6 million by 2050 estimated by the International Osteoporosis Foundation.^[3]^ Currently, surgery is still the preferred treatment for HF. Despite ongoing advancements in medical technology, care, and rehabilitation, HF patients still have a high mortality rate.^[4]^ Correa et al reported that longer length of stay (LOS) is associated with higher mortality, readmission, and increased medical costs in HF patients.^[5]^ The prolonged LOS places a significant burden on clinical care and undermines the equitable distribution of medical resources.^[6]^ Therefore, more attention should be paid to identifying LOS-related factors to strengthen HF management, promote patient recovery, and reduce LOS.

Blood urea nitrogen (BUN) is the main end product of protein metabolism, which is produced by the liver and is excreted mainly by the kidneys.^[7]^ BUN levels rise when protein is over-broken down or when the glomerular filtration rate is reduced. Therefore, BUN can reflect the catabolism of protein and is used as a biomarker to assess renal function.^[8]^ High BUN is associated with the incidence of cardiogenic shock.^[9]^ In addition, Lee et al^[10]^ reported that high BUN levels were associated with an increase in 30-day in-hospital mortality in patients with ST-elevation myocardial infarction combined with severe cardiogenic shock receiving extracorporeal membrane oxygen-assisted therapy. The upregulated admission BUN level was related to all-cause mortality in acute pulmonary embolism.^[11]^ However, there is a lack of studies on the correlation between admission BUN levels and postoperative LOS in HF.

Herein, this study aimed to analyze the relationship between admission BUN levels and LOS after surgery and explore its value in predicting first intensive care unit (ICU) admission as well as in-hospital mortality in HF patients.

## 2. Materials and methods

### 2.1. Study participants

This study was a retrospective study based on the MIMIC-IV database (https://physionet.org/content/mimiciv/2.0/). The project was approved by the Institutional Review boards of Massachusetts Institute of Technology and Beth Israel Deaconess Medical Center and was granted a waiver of informed consent.

Inclusion criteria: the 9th and 10th editions of the International Classification of Diseases (ICD-9 and ICD-10), including acetabular fracture, subtrochanteric fracture, intertrochanteric fracture, and femoral neck fracture; age ≥ 18 years old; underwent hip arthroplasty or reduction with internal fixation. Exclusion criteria: the patient did not have first HF or combined with other fractures (n = 1067); pathological fractures (n = 120), open fractures (n = 119), secondary fractures, or stress fractures (n = 13); BUN data were absent 24 hours after admission (n = 141); LOS < 24 hours (n = 71). If the patient was admitted multiple times during the study period, only the records of the first admission were selected. Finally, the study included 1274 samples.

### 2.2. Data collection

All data for this study were extracted from the MIMIC-IV database using the PostgreSQL tool (version 12.0). If the same variable is monitored multiple times after admission, only the first monitoring record for each variable is used. The following variables were collected: demographic characteristics: gender, age, body mass index (BMI); fracture condition: fracture type, surgical method; comorbidities: coronary heart disease, chronic obstructive pulmonary disease, hypertension, osteoporosis, diabetes; personal behavior history: smoking, alcohol use; laboratory tests (all taken preoperatively): bicarbonate, anion gap, total calcium, creatinine, glucose, hematocrit, hemoglobin, mean erythrocyte hemoglobin content, mean erythrocyte hemoglobin concentration, BUN, potassium, sodium, white blood cells; primary clinical outcome: LOS; Secondary clinical outcomes: ICU admission, in-hospital mortality, readmission due to fracture.

### 2.3. Statistical analysis

All data analyses were performed using SPSS software (version 25.0) and R Studio software (version 4.1.2). *P* < .05 was considered to be indicative of statistical significance.

According to BUN quartile levels, all patients were divided into 4 groups: Q1 (≤13 mg/dL, n = 334), Q2 (13 < BUN ≤ 19 mg/dL, n = 358), Q3 (19 < BUN ≤ 26 mg/dL, n = 278), Q4 (>26 mg/dL, n = 304). The categorical variables were expressed as n (%), and the comparisons between groups were evaluated using the Chi-square test. The Kolmogorov–Smirnov test was used to test the normality of continuous variables. The non-normal distribution data were represented by the median and interquartile range (IQR), and the comparisons between groups were performed by the Kruskal–Wallis *H* test. Variance inflation factor (VIF) was used to test for multicollinearity among the variables with VIF > 10 were considered as determining the presence of serious multicollinearity.

Then, generalized additive model (GAM) was employed to assess the nonlinear relationship between BUN and LOS. Three generalized linear models (GLM) were used to study the association of BUN as a continuous variable and categorical variable with LOS. Model 1: adjusted for mean erythrocyte hemoglobin concentration, creatinine, anion gap, bicarbonate, glucose, total calcium, potassium, sodium, age, and BMI; Model 2: corrected for fracture types and surgical method other than variables in model 1; Model 3: corrected for diabetes, coronary heart disease, alcohol use, and hypertension in addition to variables in model 2. A logarithmic transformation can solve the possible negative results.^[12]^ Therefore, the logarithmic transformation of LOS was performed in this study for subsequent analysis. Trend *P* value was obtained from the trend regression analysis to study the correlation between BUN trend change and LOS. Quantile regression analysis was conducted to explore the impact of BUN at different quantile levels on LOS. Subgroup analysis was performed according to clinical characteristics to further study their correlation. Interaction analysis investigated whether the relevant clinical variables were interaction factors of BUN and LOS correlation. In addition, the clinical value of BUN in predicting secondary outcomes was investigated by receiver operating characteristic curve (ROC) and decision curve analysis (DCA).

## 3. Results

### 3.1. Patient characteristics

According to the inclusion and exclusion criteria, 1274 patients (440 males and 834 females) were included, aged 19 to 91 years (average age, 79 years). The longest LOS was 76.9 days and the median LOS was 4.9 days. The BUN range was (3–129 mg/dL), with a median of 19.0 mg/dL. The patients were divided into 4 groups according to the BUN quartile levels, and the distribution of clinical features in 4 groups is shown in Table 1. The distributions of surgical method, fracture type, coronary heart disease, hypertension, diabetes, and alcohol use were different between 4 groups (*P* < .05). Older age, higher BMI, and elevated levels of anion gap, total calcium, creatinine, glucose, BUN, potassium, sodium, and white blood cells were observed in the higher BUN quartile groups (*P* < .05). However, hematocrit, hemoglobin, and mean erythrocyte hemoglobin concentration were significantly lower in the higher BUN quartile groups (*P* < .05). Other variables including chronic obstructive pulmonary disease, osteoporosis, smoking, gender, mean erythrocyte hemoglobin content, and white blood cells had no obvious changes in 4 BUN groups (*P* > .05). There were significant differences in LOS, ICU admission, and in-hospital mortality among the 4 groups, but no significant differences were observed in readmission status. To exclude the collinearity of the variables, hemoglobin and hematocrit with VIF values > 10 were removed (Table 2).

**Table 1:** Patient characteristics.

Variables		Blood urea nitrogen quartile levels				*P* value
		Q1 (n = 334)	Q2 (n = 358)	Q3 (n = 278)	Q4 (n = 304)	
Surgical method, n (%)	Hip arthroplasty	97 (29.04)	120 (33.52)	102 (36.69)	123 (40.46)	.020
	reduction with internal fixation	237 (70.96)	238 (66.48)	176 (63.31)	181 (59.54)	
Fracture type, n (%)	Acetabular fracture	22 (6.59)	16 (4.47)	7 (2.52)	8 (2.63)	.049
	Intertrochanteric fracture	89 (26.65)	106 (29.61)	104 (37.41)	103 (33.88)	
	Subtrochanteric fracture	25 (7.48)	23 (6.43)	14 (5.04)	19 (6.25)	
	Femoral neck fracture	198 (59.28)	213 (59.49)	153 (55.03)	174 (57.24)	
Coronary heart disease, n (%)	Yes	8 (2.40)	17 (4.75)	21 (7.55)	31 (10.20)	<.001
	No	326 (97.60)	341 (95.25)	257 (92.45)	273 (89.80)	
COPD, n (%)	Yes	4 (1.20)	10 (2.79)	11 (3.96)	14 (4.61)	.066
	No	330 (98.80)	348 (97.21)	267 (96.04)	290 (95.40)	
Hypertension, n (%)	Yes	139 (41.62)	199 (55.59)	161 (57.91)	106 (34.87)	<.001
	No	195 (58.38)	159 (44.41)	117 (42.09)	198 (65.13)	
Osteoporosis, n (%)	Yes	70 (20.96)	92 (25.70)	54 (19.42)	59 (19.41)	.153
	No	264 (79.04)	266 (74.30)	224 (80.58)	245 (80.59)	
Diabetes, n (%)	Yes	27 (8.08)	62 (17.32)	64 (23.02)	119 (39.15)	<.001
	No	307 (91.92)	296 (82.68)	214 (76.98)	185 (60.86)	
Alcohol use, n (%)	Yes	16 (4.79)	6 (1.68)	9 (3.24)	4 (1.32)	.025
	No	318 (95.21)	352 (98.32)	269 (96.76)	300 (98.68)	
Smoking, n (%)	Yes	38 (11.38)	34 (9.50)	39 (14.03)	42 (13.82)	.233
	No	296 (88.62)	324 (90.50)	239 (85.97)	262 (86.18)	
Gender, n (%)	Male	95 (28.44)	99 (27.65)	114 (41.01)	132 (43.42)	.892
	Female	239 (71.56)	259 (72.35)	164 (58.99)	172 (56.58)	
Age (yr)		68.00 [55.00, 80.00]	79.00 [68.00, 86.00]	82.00 [76.00, 88.00]	83.00 [73.00, 89.00]	<.001
Body mass index (kg/m^2^)		23.30 [20.80, 26.70]	24.00 [20.80, 27.90]	24.40 [21.30, 27.70]	24.70 [21.90, 29.00]	.006
Anion gap (mmol/L)		13.00 [11.00, 14.00]	13.00 [12.00, 15.00]	14.00 [12.00, 16.00]	15.00 [13.00, 17.00]	<.001
Bicarbonate (mEq/L)		26.00 [24.00, 28.00]	25.00 [24.00, 27.00]	25.00 [23.00, 27.00]	25.00 [22.00, 26.00]	<.001
Total calcium (mg/dL)		8.40 [8.10, 8.80]	8.50 [8.10, 8.90]	8.50 [8.20, 8.90]	8.60 [8.30, 9.00]	<.001
Creatinine (mg/dL)		0.70 [0.60, 0.80]	0.80 [0.70, 0.90]	1.00 [0.80, 1.20]	1.50 [1.20, 2.10]	<.001
Glucose (mg/dL)		117.00 [105.00, 136.00]	125.00 [109.00, 148.00]	121.00 [106.00, 148.00]	129.00 [110.00, 158.00]	<.001
Hematocrit (%)		33.60 [30.20, 37.00]	33.60 [30.50, 37.10]	32.50 [28.80, 37.10]	31.10 [27.80, 34.50]	<.001
Hemoglobin (g/dL)		11.10 [9.90, 12.20]	11.30 [10.00, 12.40]	10.80 [9.60, 12.20]	10.20 [9.00, 11.30]	<.001
Mean erythrocyte hemoglobin content (pg)		30.50 [29.50, 31.60]	30.50 [29.20, 31.70]	30.50 [29.20, 32.00]	30.50 [28.70, 31.80]	.602
Mean erythrocyte hemoglobin concentration (g/dL)		33.30 [32.50, 34.20]	33.10 [32.40, 34.10]	33.00 [31.8, 33.70]	32.70 [31.60,33.50]	<.001
Blood urea nitrogen (mg/dL)		11.00 [9.00, 12.00]	16.00 [15.00, 18.00]	22.00 [21.00, 24.00]	35.00 [30.00, 45.00]	<.001
Potassium (%)		4.00 [3.70, 4.20]	4.10 [3.80, 4.40]	4.20 [3.90, 4.60]	4.50 [4.10, 4.90]	<.001
Sodium (mEq/L)		138.00 [136.00, 140.00]	138.00 [136.00,140.00]	138.00 [136.00, 141.00]	139.00 [137.00, 141.00]	.007
White blood cells (K/µL)		9.50 [7.30, 11.80]	10.00 [7.60,12.50]	9.50 [7.50,12.30]	9.70 [7.70, 12.00]	.539
Length of stay (d)		4.63 [3.62, 6.23]	4.79 [3.68, 6.67]	4.83 [3.85, 6.88]	5.62 [4.34, 7.76]	<.001
ICU admission, n (%)	No	298 (89.22)	317 (88.55)	236 (84.89)	248 (81.58)	.018
	Yes	36 (10.78)	41 (11.45)	42 (15.11)	56 (18.42)	
Readmission due to fracture, n (%)	No	327 (97.90)	341 (95.25)	267 (96.04)	297 (97.70)	.158
	Yes	7 (2.10)	17 (4.75)	11 (3.96)	7 (2.30)	
In-hospital mortality, n (%)	No	333 (99.70)	356 (99.44)	270 (97.12)	290 (95.40)	<.001
	Yes	1 (0.30)	2 (0.56)	8 (2.88)	14 (4.60)	

COPD = chronic obstructive pulmonary disease, ICU = intensive care unit.

**Table 2:** Collinearity analysis.

Variables	Variance inflation factor
Hemoglobin (g/dL)	65.881
Hematocrit (%)	59.158
Mean erythrocyte hemoglobin concentration (g/dL)	4.670
Blood urea nitrogen (mg/dL)	2.355
Creatinine (mg/dL)	2.133
Anion gap (mmol/L)	1.856
Bicarbonate (mEq/L)	1.613
Surgical method	1.506
Fracture type	1.436
Age (yr)	1.345
Diabetes	1.291
Potassium (%)	1.284
Total calcium (mg/dL)	1.276
Glucose (mg/dL)	1.257
Hypertension	1.154
Sodium (mEq/L)	1.130
Coronary heart disease	1.106
Body mass index (kg/m^2^)	1.082
Alcohol use	1.048

Generalized additive model.

### 3.2. Correlation between BUN and LOS

**Figure 1.**
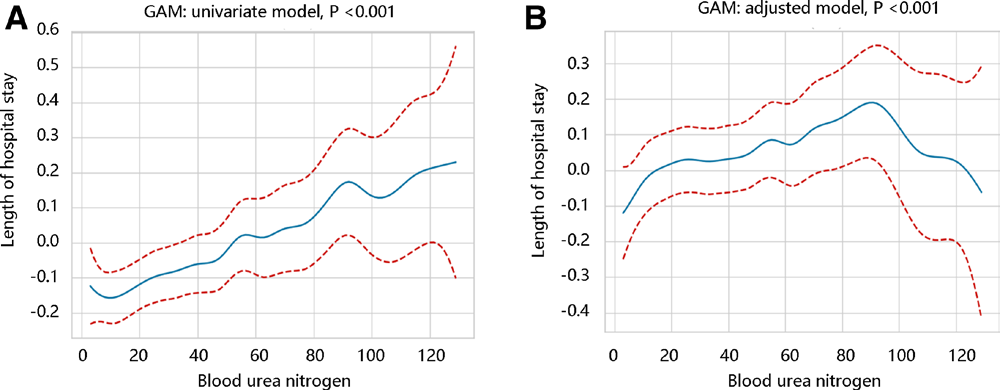
Generalized additive model analysis. There was a nonlinear relationship between blood urea nitrogen and length of stay in the (A) univariate model and (B) adjusted model.

First, GAM was employed to test the nonlinear relationship between BUN and LOS for both univariate and adjusted models (*P* < .001) (Fig. 1A and B). Due to the nonlinear relationship, GLM was adopted to assess the association of BUN with LOS. The BUN levels could independently predict the LOS after adjusting various covariates (*P* < .05). When BUN was coded into 4 groups, BUN in Q3 and Q4 were still closely related to longer LOS in different models (*P* < .05). Trend test results showed that LOS would increase significantly with the increase of BUN levels (*P* < .05) (Table 3).

**Table 3:** The association of BUN with LOS using the generalize linear models.

Variables	Model 1		Model 2		Model 3	
	Β (95% CI)	*P* value	Β (95% CI)	*P* value	Β (95% CI）	*P* value
Log LOS						
BUN levels	0.003 (0.001–0.004)	<.001	0.002 (0.001–0.004)	<.001	0.002 (0.001–0.004)	<.001
BUN quartiles						
Q1	Ref.		Ref.		Ref.	
Q2	0.027 (−0.007 to 0.061)	.120	0.026 (−0.007 to 0.060)	.121	0.028 (−0.005 to 0.061)	.100
Q3	0.057 (0.019–0.095)	.003	0.053 (0.016–0.090)	.005	0.053 (0.016–0.091)	.005
Q4	0.071 (0.028–0.113)	.001	0.065 (0.023–0.107)	.003	0.063 (0.020–0.105)	.004
*P* for trend	<.001		.001		.002	

BUN = blood urea nitrogen, LOS = length of stay, 95% CI = 95% confidence interval.

**Figure 2.**
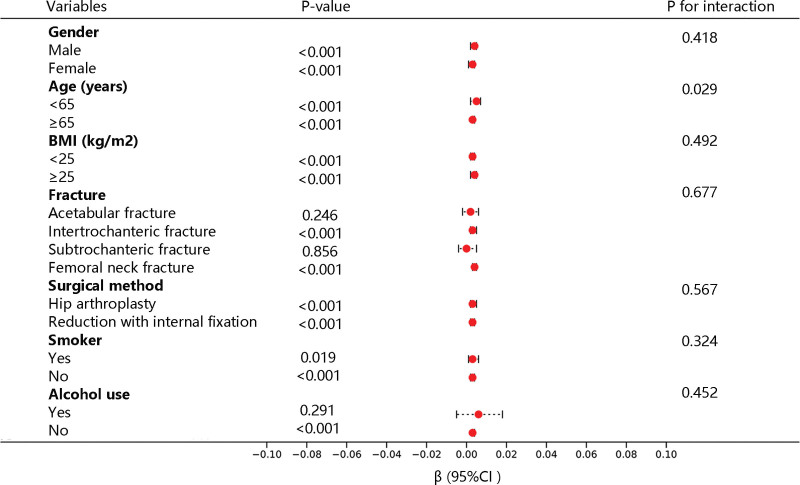
Forest plot of the association of blood urea nitrogen with length of stay in different stratifications according to clinical characteristics. BMI = body mass index.

In the subgroup analysis, BUN upregulation was still linked to longer LOS regardless of gender, age, BMI, surgical method, or smoking status (*P* < .05). Besides, the increased BUN levels were associated with increased LOS among patients with intertrochanteric fracture, femoral neck fracture, and nonpatients (*P* < .05). Among them, age played an interactive role in the correlation between BUN and LOS (Fig. 2).

**Figure 3.**
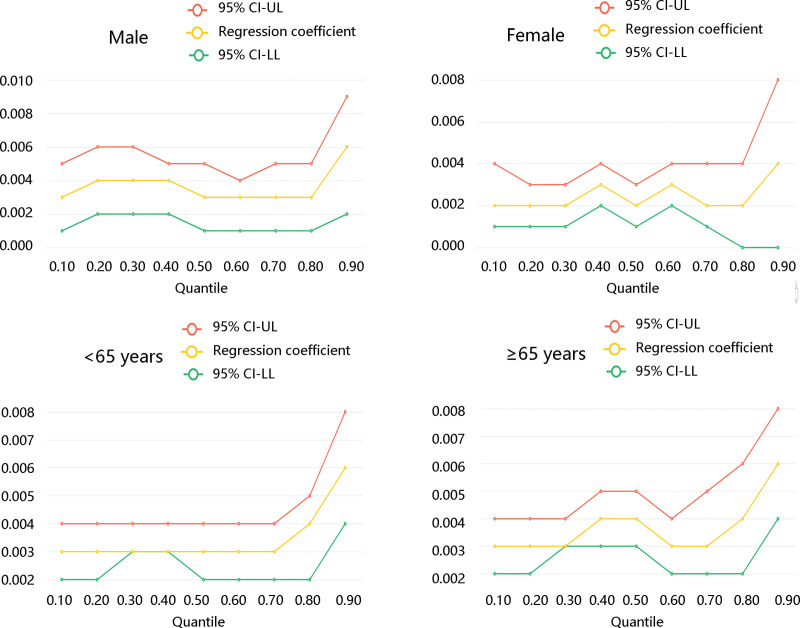
The positive association of blood urea nitrogen with length of stay stratified by age and gender. 95% CI-UL = 95% confidence interval-upper limit; 95% CI-LL = 95% confidence interval-lower limit.

To further explore the connection between BUN and LOS, quantile regression analysis was conducted. BUN at 0.1, 0.2, 0.3, 0.4, 0.5, 0.6, 0.7, 0.8, and 0.9 quantile levels were all associated with LOS in model 1 and model 2 (all *P* < .05). Except at 0.8 quantile level, BUN elevation was related to longer LOS with statistical significance (*P* < .05) (Table 4). Figure 3 demonstrated the stratification results and there still existed a positive relationship between BUN and LOS.

**Table 4:** The connection between blood urea nitrogen and length of stay using quantile regression analysis.

Quantile	Model 1	Model 2	Model 3
	Coefficients (95% CI)	Coefficients (95% CI)	Coefficients (95% CI)
0.1	0.002 (0.001–0.003)**	0.002 (0.001–0.003)***	0.002 (0.001–0.003)***
0.2	0.002 (0.001–0.003)***	0.001 (0.001–0.003)**	0.002 (0.001–0.003)***
0.3	0.002 (0.001–0.004)***	0.002 (0.001–0.004)***	0.003 (0.002–0.004)***
0.4	0.003 (0.001–0.004)***	0.002 (0.001–0.004)***	0.003 (0.001–0.004)***
0.5	0.003 (0.001–0.004)***	0.003 (0.001–0.004)***	0.003 (0.001–0.004)***
0.6	0.003 (0.001–0.004)***	0.002 (0.001–0.004)***	0.002 (0.001–0.005)***
0.7	0.002 (0.001–0.004)***	0.002 (0.001–0.004)**	0.002 (0.001–0.003)***
0.8	0.003 (0.001–0.005)***	0.002 (0.001–0.003)*	0.002 (−0.001 to 0.004)
0.9	0.006 (0.003–0.009)***	0.005 (0.002–0.007)***	0.005 (0.002–0.008)**

95% CI = 95% confidence interval.

* Indicates *P* < .05.

** Indicates *P* < .01.

*** Indicates *P* < .001.

### 3.3. Other clinical value of BUN

**Figure 4.**
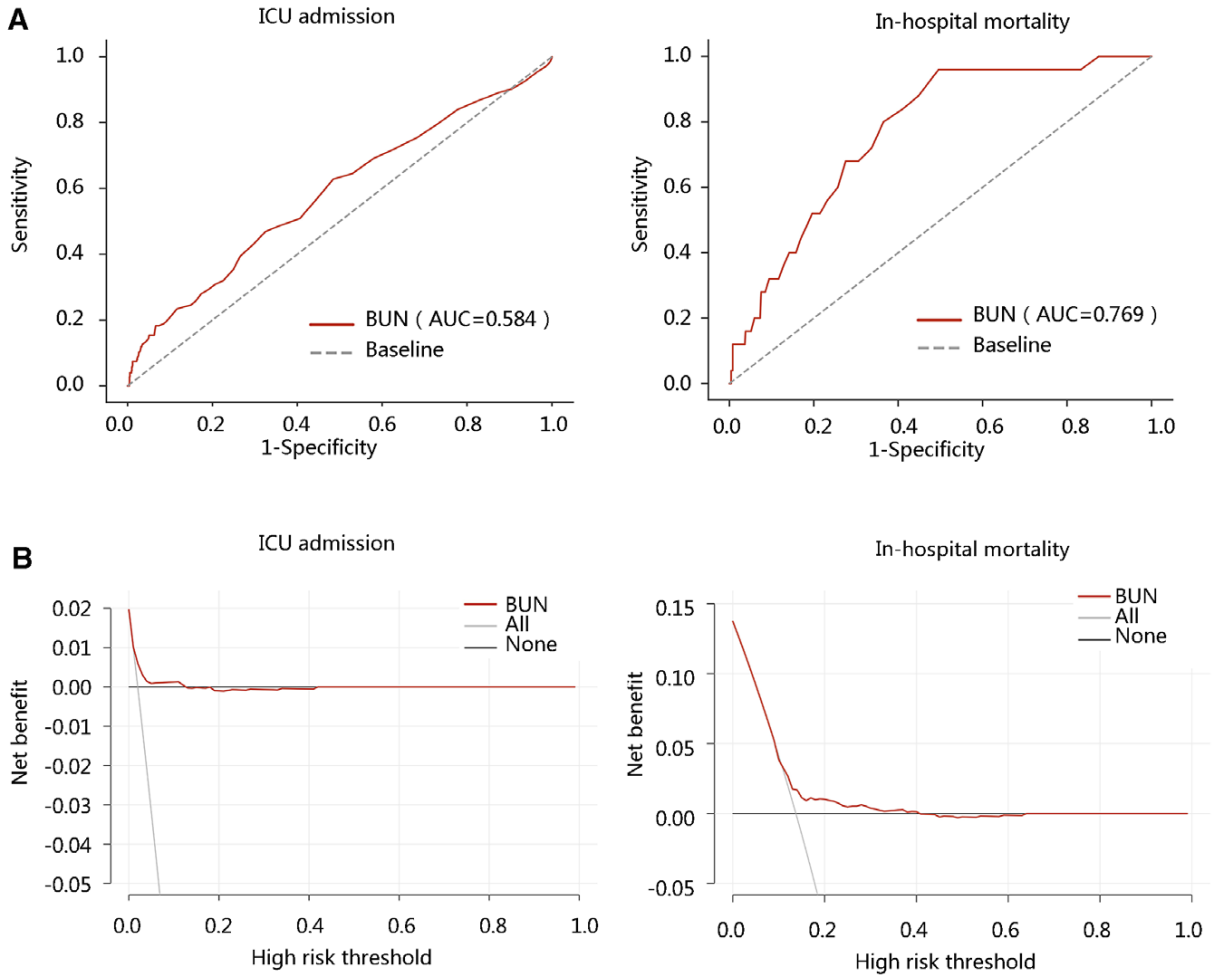
The clinical value of BUN in patients with hip fracture. (A) The receiver operating characteristic curve analysis of BUN in predicting ICU admission and in-hospital mortality. (B) The decision curve analysis revealed the clinical efficacy of BUN in terms of ICU admission and in-hospital mortality. AUC = area under the curve, BUN = blood urea nitrogen, ICU = intensive care unit.

After exploring the vital relationship between BUN and LOS, and we have demonstrated the difference in ICU admission and in-hospital mortality, we next investigated the clinical value of BUN in the 2 aspects. The area under the curve (AUC) value of the ROC was calculated, and the AUC values of BUN predicting ICU admission and in-hospital mortality were 0.584 and 0.769, respectively (Fig. 4A). The DCA results are shown in Figure 4B. These findings suggested the potential clinical value of BUN especially in predicting in-hospital mortality.

## 4. Discussion

HF is one of the most common diseases in the elderly, with high mortality, morbidity, and economic burden. Even after treatment, the life quality of patients is significantly reduced, with only 41% to 67% of patients recovering their pre-fracture walking ability within 1 year.^[13]^ Given the growth of the global elderly population, this increasing incidence of HF seems inevitable. The number of people aged ≥ 65 years was 8.5% in 2015 and is expected to rise to 12.0% by 2030 and further to 16.7% by 2050.^[14]^ Most older adults experience HF from falls, with approximately 30% of community seniors experiencing falls each year.^[15]^ Older adults are more likely to develop HF because the prevalence of osteoporosis increases with age, and older women in particular experience fall-related impairments after menopause, including dizziness, reduced balance, slow response to falls, and medical side effects.^[16]^

HF requires longer periods of recovery and rehabilitation, resulting in heavy medical costs. HF accounts for only 14% of all osteoporotic fractures but requires 72% of the total cost expenditure and is expected to cost more than $18.2 billion in the United States by 2025.^[17]^ On the one hand, bone marrow stem cells (BMSCs) are recruited to the bone surface and differentiated into osteoblasts. After mineralization, the osteoblasts are further differentiated into osteocytes. On the other hand, the binding of RANKL to the RANK receptor activates the hematopoietic stem cells and pre-precursors to enhance osteoclastogenesis in osteoporosis and bone resorption, which can be blocked by the osteoblasts-secreted osteoprotegerin. With aging, osteoblasts undergo increased apoptosis and senescence, reduced differentiation and proliferation, and dysfunctional osteoprogenitors, which result in more marrow adipogenesis, further leading to reduced BMSCs, suppressed osteoblast function, and more bone resorption.^[18–22]^ The extension of LOS has a direct impact on personal and social medical costs, as well as the increase in the rate of healthcare-associated infection, therefore, clinical work has been focused on reducing LOS.^[23]^ Different medical systems provide different perioperative care. Countries such as the United States, the United Kingdom, and Australia have a tiered system of care in which patients are discharged from a surgical hospital to an acute or subacute care facility, then to a medium or long-term care facility, and eventually discharged home or to another nonfacility. Other countries, including South Korea and Japan, provide most post-operative care and rehabilitation in surgical hospitals and discharge patients home or to other nonmedical facilities.^[13]^ Therefore, it is of great significance to find an easy-to-collect clinical indicator that can predict LOS in HF patients at an early stage.

Serum creatinine and BUN are excreted through the kidneys, and both rise with the loss of kidney function. Due to their different physiological characteristics, clinical significance is also different. Serum creatinine is produced by muscles. In critically ill patients, muscle proteolysis occurs and muscle content is reduced, resulting in a small increase in serum creatinine.^[8]^ BUN is a product of protein catabolism. Critically ill patients are in a high protein catabolic state,^[24]^ and the increase in BUN is more obvious than the elevation in serum creatinine. As expected, this study found that patients with BUN levels had longer postoperative LOS and had value in predicting postoperative LOS in patients independent of creatinine. After trauma or surgery, patients may have a series of physiological and pathological reactions caused by an inflammatory response, if excessive, it may lead to systemic inflammatory response syndrome, which may induce systemic multiple organ failure and eventually death.^[25]^ The upregulated BUN levels can reflect the inflammatory state of the kidney.^[26]^ Therefore, clinical staff need to monitor the relevant laboratory indicators, especially preoperative BUN levels, and screen high-risk groups as early as possible intervention to shorten the postoperative LOS and improve the prognosis of patients.

Subgroup analysis and interaction analysis showed that the correlation between BUN level and LOS was not limited by sex, age, BMI, surgical method, and smoking, but age was the interaction factor for this correlation. In addition, we also explored the clinical value of BUN levels in predicting ICU admission and hospitalization death by ROC and DCA analyses.

For strengths, this study was based on a large sample of the MIMIC-IV database. In addition, GLM and quantile regression analysis with 3 different models were adopted to comprehensively assess the association of BUN levels with LOS. The logarithmic transformation of LOS was performed to solve the possible negative results.

For limitations, this study may have a potential bias in data collection. A lack of standardization for laboratory parameters including blood transfusion parameters may result in a potential risk of heterogeneity between study designs and data analyses, limiting the ability to compare results and conclusions between studies. Due to the limitations inherent in the MIMIC-IV database, the findings should be validated in another independent cohorts in the future. Although the retrospective study is featured with correlation but not causality, this study lays a relevant theoretical foundation for the future application of BUN levels in predicting LOS in HF patients after surgery.

In conclusion, BUN level is significantly correlated with postoperative LOS in HF patients. Clinical intervention measures can be taken according to the preoperative BUN levels to improve the prognosis and quality of life of the patients.

## Author contributions

**Conceptualization:** Teng-di Fan.

**Data curation:** Teng-di Fan.

**Formal analysis:** Teng-di Fan.

**Writing – original draft:** Teng-di Fan, Di-kai Bei.

**Methodology:** Di-kai Bei, Qi Wang.

**Supervision:** Qi Wang.

**Writing – review & editing:** Qi Wang.

## References

[1] Maffulli N Aicale R. Proximal femoral fractures in the elderly: a few things to know, and some to forget. Medicina (Kaunas). 2022;58:1314. 36295475 10.3390/medicina58101314 PMC9612001

[2] Kanis JA Oden A McCloskey EV.; IOF Working Group on Epidemiology and Quality of Life. A systematic review of hip fracture incidence and probability of fracture worldwide. Osteoporos Int. 2012;23:2239–56. 22419370 10.1007/s00198-012-1964-3 PMC3421108

[3] Xu BY Yan S Low LL. Predictors of poor functional outcomes and mortality in patients with hip fracture: a systematic review. BMC Musculoskelet Disord. 2019;20:568. 31775693 10.1186/s12891-019-2950-0 PMC6882152

[4] Chen YP Kuo YJ Hung SW. Loss of skeletal muscle mass can be predicted by sarcopenia and reflects poor functional recovery at one year after surgery for geriatric hip fractures. Injury. 2021;52:3446–52. 34404509 10.1016/j.injury.2021.08.007

[5] Correa FG Serikawa LT Nicolau RB. Factors associated with the outcomes of older patients operated due to hip fractures. Acta Ortop Bras. 2023;31:e259371. 37151722 10.1590/1413-785220233102e259371 PMC10158960

[6] Laher AE Paruk F Richards GA. Predictors of prolonged hospital stay in HIV-positive patients presenting to the emergency department. PLoS One. 2021;16:e0249706. 33882077 10.1371/journal.pone.0249706 PMC8059827

[7] Peng R Liu K Li W. Blood urea nitrogen, blood urea nitrogen to creatinine ratio and incident stroke: the Dongfeng-Tongji cohort. Atherosclerosis. 2021;333:1–8. 34390959 10.1016/j.atherosclerosis.2021.08.011

[8] Haines RW Zolfaghari P Wan Y. Elevated urea-to-creatinine ratio provides a biochemical signature of muscle catabolism and persistent critical illness after major trauma. Intensive Care Med. 2019;45:1718–31. 31531715 10.1007/s00134-019-05760-5

[9] Oz A Cinar T Hayiroglu MI. The predictive value of plasma osmolality for in-hospital mortality in patients with acute pulmonary embolism. Clin Respir J. 2019;13:174–83. 30712325 10.1111/crj.13001

[10] Lee WC Fang CY Chen HC. Associations with 30-day survival following extracorporeal membrane oxygenation in patients with acute ST segment elevation myocardial infarction and profound cardiogenic shock. Heart Lung. 2016;45:532–7. 27601212 10.1016/j.hrtlng.2016.08.006

[11] Tatlisu MA Kaya A Keskin M. The association of blood urea nitrogen levels with mortality in acute pulmonary embolism. J Crit Care. 2017;39:248–53. 28087157 10.1016/j.jcrc.2016.12.019

[12] Verburg IW Atashi A Eslami S. Which models can I use to predict adult ICU length of stay? A systematic review. Crit Care Med. 2017;45:e222–31. 27768612 10.1097/CCM.0000000000002054

[13] Yoo J Lee JS Kim S. Length of hospital stay after hip fracture surgery and 1-year mortality. Osteoporos Int. 2019;30:145–53. 30361752 10.1007/s00198-018-4747-7

[14] Knight SP Ward M Newman L. Cardiovascular signal entropy predicts all-cause mortality: evidence from the Irish longitudinal study on ageing (TILDA). Entropy (Basel). 2022;24:676. 35626560 10.3390/e24050676 PMC9142113

[15] Veronese N Maggi S. Epidemiology and social costs of hip fracture. Injury. 2018;49:1458–60. 29699731 10.1016/j.injury.2018.04.015

[16] Solmi M Veronese N Correll CU. Bone mineral density, osteoporosis, and fractures among people with eating disorders: a systematic review and meta-analysis. Acta Psychiatr Scand. 2016;133:341–51. 26763350 10.1111/acps.12556

[17] Cao H Yu J Chang Y. Construction and validation of a risk prediction model for delayed discharge in elderly patients with hip fracture. BMC Musculoskelet Disord. 2023;24:66. 36694160 10.1186/s12891-023-06166-7 PMC9872294

[18] Jilka RL Almeida M Ambrogini E. Decreased oxidative stress and greater bone anabolism in the aged, when compared to the young, murine skeleton with parathyroid hormone administration. Aging Cell. 2010;9:851–67. 20698835 10.1111/j.1474-9726.2010.00616.x PMC2958819

[19] Farr JN Fraser DG Wang H. Identification of senescent cells in the bone microenvironment. J Bone Miner Res. 2016;31:1920–9. 27341653 10.1002/jbmr.2892 PMC5289710

[20] Abdallah BM Haack-Sorensen M Fink T. Inhibition of osteoblast differentiation but not adipocyte differentiation of mesenchymal stem cells by sera obtained from aged females. Bone. 2006;39:181–8. 16530029 10.1016/j.bone.2005.12.082

[21] Kassem M Marie PJ. Senescence-associated intrinsic mechanisms of osteoblast dysfunctions. Aging Cell. 2011;10:191–7. 21210937 10.1111/j.1474-9726.2011.00669.x

[22] Singh L Brennan TA Russell E. Aging alters bone-fat reciprocity by shifting in vivo mesenchymal precursor cell fate towards an adipogenic lineage. Bone. 2016;85:29–36. 26805026 10.1016/j.bone.2016.01.014 PMC4792752

[23] Burgers PT Van Lieshout EM Verhelst J. Implementing a clinical pathway for hip fractures; effects on hospital length of stay and complication rates in five hundred and twenty six patients. Int Orthop. 2014;38:1045–50. 24337751 10.1007/s00264-013-2218-5 PMC3997766

[24] Sharma K Mogensen KM Robinson MK. Pathophysiology of critical illness and role of nutrition. Nutr Clin Pract. 2019;34:12–22. 30580456 10.1002/ncp.10232

[25] Chen Q Hao P Wong C. Development and validation of a novel nomogram of 1-year mortality in the elderly with hip fracture: a study of the MIMIC-III database. BMJ Open. 2023;13:e068465. 10.1136/bmjopen-2022-068465 PMC10201249 37202145

[26] Wang B Lv L Wang Z. Improved anti-glioblastoma efficacy by IL-13Ralpha2 mediated copolymer nanoparticles loaded with paclitaxel. Sci Rep. 2015;5:16589. 26567528 10.1038/srep16589 PMC4645113

